# Impact of radiation on host immune system in patients treated with chemoradiotherapy and durvalumab consolidation for unresectable locally advanced non-small cell lung cancer

**DOI:** 10.3389/fonc.2023.1186479

**Published:** 2023-06-16

**Authors:** Corentin Pasquier, Léonor Chaltiel, Carole Massabeau, Audrey Rabeau, Louisiane Lebas, Amélie Lusque, Jean-Sébastien Texier, Elizabeth Cohen-Jonathan Moyal, Julien Mazières, Jonathan Khalifa

**Affiliations:** ^1^ Department of Radiation Oncology, Institut Claudius Regaud/Institut Universitaire du Cancer de Toulouse-Oncopole, Toulouse, France; ^2^ Department of Biostatistics, Institut Claudius Regaud/Institut Universitaire du Cancer de Toulouse-Oncopole, Toulouse, France; ^3^ Department of Thoracic Oncology, Centre Hospitalier Universitaire de Toulouse, Hôpital Larrey, Toulouse, France; ^4^ Department of Pulmonology, Centre Hospitalier Intercommunal des Vallées de l’Ariège (CHIVA), Saint-Jean-de-Verges, France; ^5^ Department of Nuclear Medicine, Institut Claudius Regaud/Institut Universitaire du Cancer de Toulouse-Oncopole, Toulouse, France; ^6^ Université de Toulouse III Paul Sabatier, Toulouse, France; ^7^ Institut National de la Santé et de la Recherche Médicale U1037, Centre de Recherche contre le Cancer de Toulouse, Toulouse, France

**Keywords:** radiotherapy, immunotherapy, non-small cell lung cancer (NSCLC), tumor-draining lymph nodes (TDLN), EDRIC, lymphopenia, elective node irradiation

## Abstract

**Background:**

The optimal modalities of radiotherapy when combining concurrent chemoradiation (CCRT) and immunotherapy (IO) for locally advanced non-small cell lung cancer (LA-NSCLC) remain to be determined. The aim of this study was to investigate the impact of radiation on different immune structures and immune cells in patients treated with CCRT followed by durvalumab.

**Material and methods:**

Clinicopathologic data, pre- and post-treatment blood counts, and dosimetric data were collected in patients treated with CCRT and durvalumab consolidation for LA-NSCLC. Patients were divided into two groups according to the inclusion (NILN-R+) or not (NILN-R−) of at least one non-involved tumor-draining lymph node (NITDLN) in the clinical target volume (CTV). Progression-free survival (PFS) and overall survival (OS) were estimated by the Kaplan–Meier method.

**Results:**

Fifty patients were included with a median follow-up of 23.2 months (95% CI 18.3–35.2). Two-year PFS and 2-year OS were 52.2% (95% CI 35.8–66.3) and 66.2% (95% CI 46.5–80.1), respectively. In univariable analysis, NILN-R+ (hazard ratio (HR) 2.60, p = 0.028), estimated dose of radiation to immune cells (EDRIC) >6.3 Gy (HR 3.19, p = 0.049), and lymphopenia ≤ 500/mm^3^ at IO initiation (HR 2.69, p = 0.021) were correlated with poorer PFS; lymphopenia ≤ 500/mm^3^ was also associated with poorer OS (HR 3.46, p = 0.024). In multivariable analysis, NILN-R+ was the strongest factor associated with PFS (HR 3.15, p = 0.017).

**Conclusion:**

The inclusion of at least one NITDLN station within the CTV was an independent factor for poorer PFS in the context of CCRT and durvalumab for LA-NSCLC. The optimal sparing of immune structures might help in achieving better synergy between radiotherapy and immunotherapy in this indication.

## Introduction

The most important improvement in patients with unresectable stage III non-small cell lung cancer (NSCLC) was recently obtained by the addition of consolidation immunotherapy (durvalumab) to concurrent chemoradiation (CCRT), which now constitutes the standard of care (1, 2). However, the optimal radiation therapy regimen in the context of immunotherapy remains to be determined.

One of the immunosuppressive effects of radiotherapy is the direct depletion of circulating lymphocytes or progenitors in lymphoid organs (3–5). Before the era of immunotherapy (IO), several studies highlighted the detrimental impact of lymphopenia on patients treated by CCRT for unresectable LA-NSCLC (5–8). Several models have been proposed to estimate the dose delivered to circulating immune cells. Jin et al. developed a three-step model to calculate the estimated dose of radiation to immune cells (EDRIC) during thoracic radiotherapy, assuming the following: a) the dose to circulating immune cells including rapidly circulating ones in the heart, lung, and blood vessels and slowly circulating ones in the lymphatic system and blood reservoirs (a portion of veins/capillaries) is a surrogate for the EDRIC; b) at each fraction, the radiation dose is uniformly delivered to all cells for rapidly circulating ones and only to those in the irradiated volume for slowly circulating cells. In this model, the blood dose relating to the contribution of a given organ is approximated by its mean organ dose, the percentage of cardiac output, the percentage of blood volume it receives, the time for one blood circulation, the irradiation time, and the number of fractions. Second, the equivalent uniform dose (EUD) is determined from a blood dose/volume histogram (percentage of blood volume irradiated at a given dose). Third, the EDRIC is the sum of the EUDs of each organ. In summary, the EDRIC can be approximated as a function of the mean heart dose, the mean lung dose, the mean body dose, and the number of fractions (9). In a secondary analysis of the RTOG 0617 trial, they showed that a higher EDRIC was significantly associated with poorer outcomes (10). The model was adjusted and externally validated in a retrospective cohort of stage III NSCLC following definitive CCRT (11). In addition, EDRIC was negatively associated with lymphocyte and neutrophil counts. More recently, the impact of lymphopenia (12–14) and EDRIC (15) on outcomes has been suggested in the context of durvalumab consolidation.

As the tumor-draining lymph nodes (TDLNs) are the main sites of lymphocyte priming, their sparing in the context of radiotherapy and IO should be addressed. In the context of high dose per fraction, preclinical models have established the deleterious effect of TDLN irradiation on the radiation-induced anti-tumor immune response, whether or not it is associated with immune checkpoint inhibitors (16–18). However, the impact of radiation dose on non-involved tumor-draining lymph nodes (NITDLNs) as well as other “immune” organs at risk (iOARs), such as bone marrow, spleen, and immune cells in the context of conventionally fractionated CCRT and durvalumab consolidation for stage III NSCLC, remains to be established.

Therefore, the aim of this study was to assess the impact of radiation on different immune structures including NITDLNs and iOARs with regard to clinical outcomes in a cohort of patients treated by CCRT followed by durvalumab.

## Materials and methods

### Study population

Between January 2015 and March 2022, patients with unresectable LA-NSCLC who underwent platinum-based CCRT followed by durvalumab consolidation were retrospectively analyzed through the electronic database of a Comprehensive Cancer Center (xxx). Inclusion criteria were as follows: 1) histologically documented NSCLC; 2) imaging evaluation including at least computed tomography (CT) of the chest, abdomen, and pelvis and/or F-18 fluorodeoxyglucose positron emission tomography/computed tomography (^18^F FDG-PET/CT) and brain CT or magnetic resonance imaging (MRI); 3) diagnosis of unresectable locally advanced disease; 4) treatment with platinum-based CCRT (at least two cycles concurrent with radiotherapy) and initiation of durvalumab consolidation therapy (10 mg/kg every 2 weeks or 1,500 mg every 4 weeks) if no disease progression after CCRT; 5) complete blood counts accessible at baseline and follow-up. Patients who underwent sequential chemoradiotherapy were excluded.

This study was approved by the institutional review boards of our institution. Patients received a letter detailing the aim of the study and the use of data collection and could refuse inclusion at any time, but informed consent was not necessary because of the retrospective nature of the study.

### Data collection

#### Demographics

Patient characteristics such as age, WHO performance status, clinical staging (TNM), histology, PD-L1 expression, and mutational status were collected. Standardized uptake value (SUV) max and SUV peak were evaluated from ^18^F FDG-PET/CT of eligible patients by a single physician.

#### Radiotherapy data

Treatment was delivered by intensity-modulated radiation therapy (IMRT) with volumetric modulated arc therapy (VMAT), and motion was managed by using 4D-CT and motion-adapted gross tumor volume (GTV) in all patients. Target volumes for the whole cohort were based on the European guidelines (19–21). The lung window setting on planning CT scan was used to delineate the GTV of the primary tumor. Depending on the histology, a 5–8-mm expansion was made and edited accounting for the surrounding anatomy to create the clinical target volume (CTV) of the primary tumor. Lymph nodes were included in target volumes in the event of enlarged and/or FDG-avid nodes (21). Nodal CTV was defined as the whole lymph node station(s) of the involved node(s). A 5-mm margin was applied around the CTV to create the planning target volume (PTV) in all patients. Patients were treated with curative intent radiotherapy, most commonly to 66 Gy in 2-Gy fractions, prescribed on the median of the PTV.

NITDLNs were retrospectively segmented in eligible patients by a single physician. Lymph node stations were defined according to the Japan Lung Cancer Society atlas (22). TDLNs were defined according to the topography of the primary tumor (23–25): stations 10/11R, 7, 4R, and 2R for right upper lobe tumors; stations 10/11R, 8, 7, 4R, and 2R for middle lobe or right lower lobe tumors; stations 10/11L, 7, 2L, 4L, 5, and 6 for left upper lobe tumors; stations 10/11L, 7, 2L, 4L, 5, 6, and 8 for left lower lobe tumors.

Patients were divided into two groups: 1) patients with at least one NITDLN station included in the CTV (radiation to non-involved lymph node (NILN-R+)) and 2) patients with no NITDLN station included in the CTV (NILN-R−).

In addition, we retrospectively delineated the thoracic vertebrae from T1 to T12 and the spleen on planning CT scans.

All dosimetric parameters were extracted from dose–volume histograms available in our planning system (Eclipse^®^, Varian, Palo Alto, CA, USA).

#### Biological data

White blood cell (WBC) count, absolute lymphocyte count (ALC), absolute neutrophil count (ANC), and neutrophil-to-lymphocyte ratio (NLR) were collected from complete blood count before treatment, at the end of CCRT and the initiation of consolidative immunotherapy (durvalumab). ALC nadir was also reported. Lymphopenia was graded according to the Common Terminology Criteria for Adverse Events (CTCAE v5.0). A cutoff of 500/mm^3^ was chosen as clinically relevant in this cohort.

The lymphocyte variation rate (LVR) from baseline to the end of CCRT was calculated according to the following equation:


$$\frac{\left(ALC\;end\;CCRT-ALC\;baseline\right)}{ALC\;baseline}\;\times \;100.$$

#### Calculation of EDRIC

EDRIC was calculated by using dosimetric data including mean heart dose (MHD), mean lung dose (MLD), mean body dose (MBD), and the number of fractions as reported by Ladbury et al. (11):


$$EDRIC=0.12\times MLD+0.08\times MHD+\;\left[0.45\;+\;0.35\times 0.85\times {\left(\frac{\#of\;fractions}{45}\right)}^{1/2}\right]\times MBD.$$

With the use of data from Jin et al. and Ladbury et al. (10, 11), the 6.3-Gy cutoff was used to split the cohort into two groups.

### Outcomes and follow-up

Overall survival (OS) was defined as the time from initiation of durvalumab to death or the last follow-up (censored data). Progression-free survival (PFS) was defined as the time from initiation of durvalumab to progression or death. Patients still alive and without recurrence were censored at the last follow-up. Time to local recurrence (TLR) was defined as the time from initiation of durvalumab to local recurrence. Patients who did not experience local recurrence as the first event were censored at the date of the first event (distant recurrence or death) or the last follow-up.

Controlled disease after CCRT was confirmed by CT in all patients. During consolidation IO, patients were monitored by full-body CT scan and clinical examination every 3 months. At the end of durvalumab or after confirmation of the first disease progression, follow-up was at the discretion of the treating oncologist.

Tumor response was evaluated according to the Response Evaluation Criteria in Solid Tumors (RECIST), version 1.1 (26).

### Statistical analysis

Data were summarized by frequency and percentage for qualitative variables and by median and range for continuous variables. Groups were compared by using the chi-square or Fisher’s exact test for qualitative variables and the Kruskal–Wallis test for continuous variables. Correlations between continuous variables were calculated with Spearman’s coefficient.

All survival times were estimated by the Kaplan–Meier method with 95% confidence intervals (CIs). In univariable analyses, p-values were calculated by using the Cox proportional hazards model for continuous variables and the log-rank test for qualitative variables, and hazard ratios (HRs) with 95% confidence intervals were estimated with the Cox proportional hazards model for each variable. Cox proportional hazards model was also used to perform multivariable analyses. HRs with 95% confidence intervals were estimated for each variable.

All statistical tests were two-sided, and p-values<0.05 were considered significant. No adjustment was made for multiple comparisons. Statistical analyses were conducted by using Stata^®^ version 16.

## Results

### Patient characteristics

In total, 50 patients were included. The baseline and treatment characteristics of the population are summarized in **Table 1**. Of the patients, 52% (n = 26/50) had adenocarcinoma histology, and 72% (n = 36) had stage IIIA or IIIB disease. PD-L1 expression was ≥1% in 37 patients. Most patients were treated with carboplatin-based concurrent chemoradiotherapy (58%, n = 29), and the median number of cycles of chemotherapy was 4 (range, 3–6). All patients were treated with IMRT and image-guided radiation therapy (IGRT). The median dose to the PTV was 66 Gy (55–66 Gy) with a median dose per fraction of 2 Gy (2–2.75 Gy). Eleven patients (22%) had NILN-R+. The median number of irradiated non-involved station(s) was 1 (1–3), and the most common stations targeted were 7, 4, and 2. Forty-two patients (84%) had a partial response at the end of CCRT. All patients received durvalumab as consolidative radiotherapy, and it was started at a median time of 34 days from the end of CCRT.

**Table 1:** Patients characteristics.

Baseline characteristics		Total / N =50	NILN-R- n = 39	NILN-R+ n = 11	p-value
**Age at initial diagnosis**	Median, years (Range)	61,5 (36 – 75)	61 (36-73)	68 (49-75)	0.497
**Sex**	Male Female	38 (76%) 12 (24%)	30 (77%) 9 (23%)	8 (73%) 3 (27%)	1
**Smoking history**	Current Former Never	12 (24%) 36 (72%) 2 (4%)	9 (23%) 29 (74%) 1 (3%)	3 (27%) 7 (64%) 1 (9%)	0.438
**History of previous neoplasia**	Yes No	10 (20%) 40 (80%)	8 (20%) 31 (80%)	2 (18%) 9 (82%)	1
**ECOG PS**	0 1	18 (36%) 32 (64%)	14 (36%) 25 (64%)	4 (36%) 7 (64%)	1
**Tumor histology**	Adenocarcinoma Squamous cell Other	26 (52%) 21 (42%) 3 (6%)	21 (54%) 15 (39%) 3 (7%)	5 (46%) 6 (54%) 0	0.769
**PDL1 Expression**	< 1% ≥ 1% Unknown	6 (12%) 37 (74%) 7 (14%)	5 (13%) 28 (72%) 6 (15%)	1 (9%) 9 (82%) 1 (9%)	1
**Overall stage (AJCC 8th)**	II IIIA IIIB IIIC	3 (6%) 19 (38%) 17 (34%) 11 (22%)	2 (5%) 12 (31%) 15 (39%) 10 (25%)	1 (9%) 7 (64%) 2 (18%) 1 (9%)	0.186
**Chemotherapy regimen**	Carboplatin + Vinorelbine Cisplatin + Vinorelbine Carboplatin + Pemetrexed Cisplatin + Pemetrexed	28 (56%) 19 (38%) 1 (2%) 2 (4%)	21 (54%) 16 (41%) 1 (2%) 1 (2%)	3 (27%) 7 (64%) 1 (9%) 0	0.529
**Radiation total dose and regimen**	66 Gy / 33 fx 64 Gy / 32 fx 62 Gy / 31 fx 60 Gy / 30 fx 55 Gy / 20 fx	34 (68%) 5 (10%) 1 (2%) 8 (16%) 2 (4%)	28 (72%) 3 (7%) 1 (3%) 6 (15%) 1 (3%)	6 (55%) 2 (18%) 0 (0%) 2 (18%) 1 (9%)	0.297
**Duration of CCRT**	Median, days (range)	86 (52 – 147)	85 (52 – 147)	92 (71 – 130)	0.468
**Time interval between CCRT and durvalumab initiation**	Median, days (range)	34 (6 – 81)	34 (13 – 81)	34 (6 – 54)	0.504
**Response to CCRT**	Stable disease Partial response	8 (16%) 42 (84%)	6 (15%) 33 (85%)	2 (18%) 9 (82%)	1

CCRT, concurrent chemoradiotherapy.

### Dosimetric data

In the whole cohort, the median total PTV was 326 cm^3^ (114.1–1,284 cm^3^); mean heart, lung PTV, and body dose were 9.4 Gy (0.8–17.9), 12.2 Gy (5.6–19.2), and 7.1 Gy (2.7–12), respectively. Median EDRIC was 7.6 Gy (2.8–11.6). Of the patients, 72% (36/50) had EDRIC > 6.3 Gy. Forty-two patients (84%) had NITDLNs available for dosimetric analysis because eight patients in the NILN-R− group had all the TDLNs involved. The median mean dose to NITDLNs was 40.4 Gy (25.8–64.3) in the NILN-R+ group *vs.* 23.2 Gy (3.1–58.9) in the NILN-R− group (p = 0.002).

Dosimetric data are summarized in **Table 2**.

**Table 2:** Dosimetric data.

Dosimetric parameters	Total (N = 50)	NILN-R- (N=39)	NILN-R+ (N=11)	p-value
**Volume of PTV** **(median, cm^3^) (range)**	326 (114.1 – 1284)	352.2 (114.1 – 1284)	305.8 (203.3 – 698)	0.824
**Volume of tumor GTV** **(median, cm3), (range)**	52.0 (0.4 - 837.5)	47.5 (0.4 – 837.5)	60.3 (2.4 – 266.5)	0.7
**Mean heart dose** **(median, Gy) (range)**	9.4 (0.8 – 17.9)	8.2 (0.8 – 17.9)	10.7 (1.7 – 15)	0.787
**Mean lung dose** **(lung minus PTV)** **(median, Gy) (range)**	12.2 (5.6 – 19.2)	12.6 (5.6 – 19.2)	11.2 (8.4 – 16.4)	0.218
**Mean spleen dose** **(median, Gy) (range)**	0.3 (0 – 2.8)	0.3 (0 – 2.8)	0.3 (0.1 – 1.7)	0.911
**Mean dose to T1-T12** **(median, Gy) (range)**	11.1 (2.8 – 22.6)	11.4 (2.8 – 22.5)	10.9 (5.4 – 16.3)	0.386
**Mean body dose** **(median, Gy) (range)**	7.1 (2.7 – 12)	7.3 (2.7 – 12)	6.2 (3.9 – 10.5)	0.467
**EDRIC** **(median, Gy) (range)**	7.6 (2.8 – 11.6)	7.8 (2.8 – 11.6)	6.8 (4.2 – 10.6)	0.355
**EDRIC** **≤ 6.3 Gy (n, %)** **> 6.3 Gy (n, %)**	14 (28%) 36 (72%)	10 (26%) 29 (74%)	4 (36%) 7 (64%)	0.475
**Volume NITDLN** **(median, cm^3^) (range)**	26.5 (0 – 132.1)	26.3 (0 – 132.1)	31 (6.5 – 90.5)	0.297
***Dose NITDLN***	*Total (N =41)*	*NILN-R- (N=31)*	*NILN-R+ (N=11)*	*p-value*
**Mean dose Gy** **(median, Gy) (range)**	28.9 (3.1 – 64.3)	23.2 (3.1 – 58.9)	40.4 (25.9 – 64.3)	0.002
**V10Gy %, (range)**	76.4 (2.7 – 100)	74.2 (2.7 – 100.0)	89.9 (43.8 – 100.0)	0.112
**V20Gy %, (range)**	59.3 (0.0 – 100)	51.7 (0.0 – 100.0)	76 (42 – 100.0)	0.014
**V30Gy %, (range)**	40.3 (0.0 – 100)	36.4 (0.0 – 99.8)	66.7 (34.4 – 100)	0.006
**V40Gy %, (range)**	31.1 (0.0 – 100)	18.9 (0.0 – 98.4)	57 (19.8 – 100)	0.001
**V50Gy %, (range)**	20.1 (0.0 – 100)	13.5 (0.0 – 92.7)	49.2 (13.2 – 100)	0.001

NILN-R+, inclusion of at least one non-involved tumor draining lymph node; NITDLN, non-involved tumor draining lymph node; EDRIC, estimated dose to immune cells; ALC, absolute lymphocyte count.

### Biological data

Median ALC at baseline, at the end of CCRT, and IO initiation was 1,715/mm^3^ (628–3,060), 495/mm^3^ (130–1,500), and 705/mm^3^ (175–1,960), respectively. Median NLR at baseline, at the end of CCRT, and IO initiation was 2.75 (0.9–10.5), 5.46 (2–24.6), and 4.28 (1.4–16.2), respectively. Eleven patients (22%) experienced lymphopenia ≤ 500/mm3 at IO initiation. Median nadir lymphopenia was 480/mm3 (130–1,215). Twenty-seven (54%) patients experienced grade 3 or 4 (G3/4) lymphopenia. The median LVR was −71.7% (−84.6%; −26.7%). No clinical/dosimetric difference was found between patients with ALC ≤ 500/mm^3^ and patients with ALC > 500 mm^3^ at IO initiation. Patients with ALC ≤ 500/mm^3^ at IO initiation had significantly lower ALC at baseline and the end of CCRT when compared to patients with ALC >500 mm^3^, but both groups had a similar LVR (**Supplementary Table 1**). Similarly, no clinical/dosimetric difference was found between patients with grade 1/2 lymphopenia at nadir and patients with grade 3/4 lymphopenia.

Spearman’s correlation was weak between the LVR and any clinical/dosimetric relevant variables (**Supplementary Table 2**). There was no association between the LVR and the number of concurrent chemotherapy cures (LVR of −71.7% (−84.6; −26.7) versus –71.7% (−84.3; −42.2) for 1–2 versus 3 cures, respectively) nor between the LVR and the total prescribed dose (LVR of –71.7% (−84.3; −29.5) versus −71.7% (−84.6; −26.7) for dose< 66 Gy versus dose of 66 Gy, respectively).

### Survival outcomes

The median follow-up time was 23.2 months (95% CI 18.3–35.2 months).

Fourteen patients (28%) had died at the cutoff date for analysis. The median OS was not reached, and the 2-year OS was 66.2% (95% CI 46.5–80.1). In univariable analysis, previous history of neoplasia (HR 4.44, 95% CI 1.27–15.5, p = 0.011) and lymphopenia ≤ 500/mm^3^ at IO initiation (HR 3.46, 95% CI 1.10–10.87, p = 0.024**)** were significantly associated with poor OS. In multivariable analysis, the results were similar, but the associations were not significant (**Table 3**).

**Table 3:** Univariable and multivariable analyses for overall survival.

Variable		Univariable		Multivariable	
		HR (95% CI)	p-Value	HR (95% CI)	p-Value
Sex	Male	1			
	Female	0.51 (0.11–2.29)	0.371		
ECOG PS	0	1			
	1	1.40 (0.44–4.50)	0.569		
Smoking history	Former smoker	1			
	Current smoker	1.55 (0.51–4.66)	0.436		
History of previous neoplasia	No	1		1	
	Yes	4.44 (1.27–15.50)	0.011	3.0 (0.75–11.95)	0.119
Histology	Squamous cell	1			
	Adenocarcinoma	0.42 (0.15–1.20)	*0.096*		
Overall stage (AJCC 8th)	II/IIIA/IIIB	1			
	IIIC	2.07 (0.61–7.01)	0.230		
Radiation total dose	<66 Gy	1			
	66 Gy	0.86 (0.27–2.79)	0.807		
NILN-R+	No	1			
	Yes	0.95 (0.21–4.29)	0.943		
Response to CCRT	Partial response	1			
	Stable disease	1.83 (0.57–5.86)	0.303		
Grade lymphopenia (nadir)	1/2	1			
	3/4	2.54 (0.80–8.01)	0.103		
Lymphopenia at IO initiation	>500/mm^3^	1			
	≤500/mm^3^	3.46 (1.10–10.87)	0.024	2.38 (0.66–8.56)	0.183
EDRIC	≤6.3 Gy	1			
	>6.3 Gy	2.79 (0.61–12.73)	0.168		
Age*		1.07 (0.99–1.15)	*0.093*		
SUVmax*		1.03 (0.99–1.08)	0.154		
Duration of CCRT (days)*		1.00 (0.98–1.03)	0.746		
PTV*		1.18 (0.98–1.41)	*0.073*		
Mean NITDLN dose*		1.01 (0.96–1.06)	0.708		
Days from CCRT to IO*		0.99 (0.95–1.04)	0.803		
ALC at IO initiation*		0.81 (0.63–1.04)	0.095		
Nadir lymphopenia (/mm^3^)*		0.72 (0.51–1.02)	*0.062*		
NLR at baseline*		0.98 (0.76–1.27)	0.877		
NLR at end of CCRT*		1.09 (0.97–1.22)	0.143		
NLR at IO initiation*		1.12 (0.95–1.33)	0.173		

NILN-R+, inclusion of at least one non-involved tumor-draining lymph node; NITDLN, non-involved tumor-draining lymph node; CCRT, concurrent chemoradiotherapy; IO, immunotherapy; EDRIC, estimated dose to immune cells; ALC, absolute lymphocyte count; NLR, neutrophil-to-lymphocyte ratio; ECOG PS, Eastern Cooperative Oncology Group Performance Status; AJCC, American Joint Committee on Cancer; PTV, planning target volume; SUV, standardized uptake value.

*Continuous variables.

**Figure 1:**
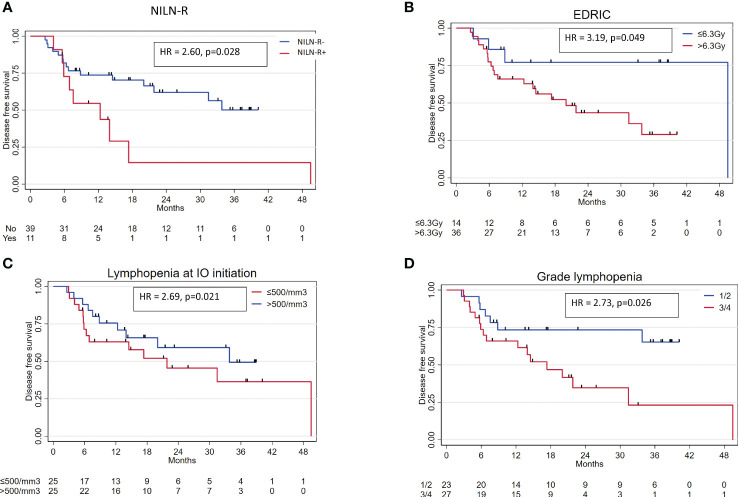
Main variables associated with PFS in univariable analysis. **(A)** NILN-R. **(B)** EDRIC. **(C)** Lymphopenia at IO initiation. **(D)** Grade of lymphopenia at nadir. NILN-R+, inclusion of at least one non-involved tumor-draining lymph node; IO, immunotherapy; EDRIC, estimated dose to immune cells; PFS, progression-free survival.

At the time of analysis, 24 patients experienced disease progression or death. The median PFS was 31.4 months (95% CI 14.0–not reached). NILN-R+ (HR 2.60, 95% CI 1.08–6.27, p = 0.028), G3/4 lymphopenia at nadir (HR 2.73, 95% CI 1.09–6.82, p = 0.026), lymphopenia ≤ 500/mm^3^ at IO initiation (HR 2.69, 95% CI 1.12–6.46, p = 0.021), and EDRIC > 6.3 Gy (HR 3.19, 95% CI 0.94–10.82, p = 0.049) were associated with worse PFS in univariable analysis (**Figure 1**). In the univariable Cox regression model for continuous variables, tumor SUVmax (HR 1.04, 95% CI 1.00–1.08, p = 0.030), ALC at IO initiation (HR 0.85, 95% CI 0.72–1.00, p = 0.047), and nadir ALC (HR 0.79, 95% CI 0.63–0.99, p = 0.038) were significantly associated with PFS. The multivariable analysis, including NILN-R+, lymphopenia ≤ 500/mm^3^ at IO initiation, EDRIC > 6.3 Gy, and SUVmax, revealed that NILN-R+ was the strongest factor associated with PFS (HR 3.15, 95% CI 1.23–8.10, p = 0.017). SUVmax was still a prognostic factor (p = 0.038), and there was a trend toward worse PFS with EDRIC > 6.3 Gy (HR 3.03, 95% CI 0.83–11.00, p = 0.093) (**Table 4**).

**Table 4:** Univariable and multivariable analyses for progression-free survival.

Variable		Univariable		Multivariable	
		HR (95% CI)	p-Value	HR (95% CI)	p-Value
Sex	Male	1			
	Female	1.25 (0.49–3.17)	0.642		
ECOG PS	0	1			
	1	2.04 (0.80–5.18)	0.127		
Smoking history	Former smoker	1			
	Current smoker	1.78 (0.72–4.38)	0.205		
History of previous neoplasia	No	1			
	Yes	1.76 (0.64–4.89)	0.269		
Histology	Squamous cell	1			
	Adenocarcinoma	0.77 (0.33–1.78)	0.540		
Overall stage (AJCC 8th)	II/IIIA/IIIB	1			
	IIIC	2.24 (0.90–5.57)	*0.076*		
Radiation total dose	<66 Gy	1			
	66 Gy	0.74 (0.31–1.77)	0.500		
NILN-R+	No	1			
	Yes	2.60 (1.08–6.27)	0.028	3.15 (1.23–8.10)	0.017
Response to CCRT	Partial response	1			
	Stable disease	1.53 (0.57–4.14)	0.398		
Grade lymphopenia (nadir)	1/2	1			
	3/4	2.73 (1.09–6.82)	0.026		
Lymphopenia at IO initiation	>500/mm^3^	1			
	≤500/mm^3^	2.69 (1.12–6.46)	0.021	1.93 (0.77–4.83)	0.158
EDRIC	≤6.3 Gy	1			
	>6.3 Gy	3.19 (0.94–10.82)	0.049	3.03 (0.83–11.0)	*0.093*
Age*		1.04 (0.98–1.09)	0.182		
SUVmax*		1.04 (1.00–1.08)	0.030	1.05 (1.00–1.09)	0.038
Duration CCRT (days)*		1.01 (0.99–1.03)	0.350		
PTV*		1.06 (0.92–1.23)	0.426		
Mean NITDLN dose*		1.02 (0.99–1.06)	0.125		
Days from CCRT to IO*		0.99 (0.96–1.02)	0.507		
ALC at IO initiation*		0.85 (0.72–1.00)	0.047		
Nadir lymphopenia (/mm^3^)*		0.79 (0.63–0.99)	0.038		
NLR at baseline*		1.14 (0.93–1.39)	0.200		
NLR at end of CCRT		1.06 (0.98–1.14)	0.155		
NLR at IO initiation		1.06 (0.94–1.20)	0.346		

NILN-R+, inclusion of at least one non-involved tumor-draining lymph node; NITDLN, non-involved tumor-draining lymph node; CCRT, concurrent chemoradiotherapy; IO, immunotherapy; EDRIC, estimated dose to immune cells; ALC, absolute lymphocyte count; ECOG PS, Eastern Cooperative Oncology Group Performance Status; NLR, neutrophil-to-lymphocyte ratio; AJCC, American Joint Committee on Cancer; PTV, planning target volume; SUV, standardized uptake value.

*Continuous variables.

The association between PD-L1 status and OS or PFS was not tested owing to the small number of PD-L1-negative patients (n = 6).

### Toxicity

At the end of CCRT, 96% had experienced at least one adverse event of any cause and grade, and 20% (10/50) had grade 3 toxicity. The most common grade 3 adverse event was hematologic toxicity (90%, 9/10), and one patient experienced grade 3 esophagitis. Immune-related adverse events (iRAEs) occurred in 66% (33/50) of patients. No grade 4 or 5 iRAE was reported. Five patients (15.2%) experienced grade 3 iRAE: three cases of musculoskeletal toxicity and two cases of skin toxicity (rash). No grade 3 pneumonitis adverse event occurred in the cohort.

## Discussion

In this retrospective study, we evaluated the impact of radiation on the immune system, in the context of CCRT followed by durvalumab for stage III NSCLC. One of the main findings is the negative effect of the prophylactic radiation of at least one NITDLN station(s) (NILN-R+). To our knowledge, this is the first study to demonstrate the deleterious impact of radiation on NITDLNs on outcomes in the era of durvalumab after CCRT in NSCLC. Indeed, radiation to NITDLNs was an independent factor for worse PFS in our cohort.

Before the era of durvalumab, involved-field radiotherapy (IFRT) in patients treated with conformational 3D radiotherapy (3D-CRT) was shown to be non-inferior to prophylactic irradiation of all NITDLN stations, known as “elective nodal irradiation” (ENI), in terms of loco-regional recurrence (27–30). In a recent randomized trial, Nestle et al. compared two target volume delineation strategies: one strategy based upon ^18^F FDG-PET/CT only versus another combining ^18^F FDG-PET/CT and CT data plus ENI. This trial was the first to show the non-inferiority of reducing target volumes and avoiding ENI based on modern molecular imaging staging (31). Moreover, the risk of locoregional progression was lower in the ^18^F FDG-PET/CT-based target group (14% *vs.* 29% at 1 year, HR 0.57; per protocol analysis).

However, it was shown that incidental dose to NITDLNs is high when using IFRT with the 3D-CRT technique, as most of the uninvolved nodal stations receive more than 40 Gy (32). Since the implementation of IMRT, there has been no formal comparison of ENI *vs.* IFRT. In the study by Nestle et al., as many as 50% of patients were treated with IMRT (31). In our cohort of IMRT-only treatment, a subgroup of patients had at least one NITDLN station included in the CTV (e.g., in the event of NITDLNs between two involved nodal stations). Most of them had only one station included (64%), and the most common stations targeted were stations 2, 4, and 7. Whether or not this strategy is safe in the context of IMRT and consolidation IO remained to be established; herein, we showed that NILN-R+ was associated with worse outcomes.

These findings are in line with a disturbance of the anti-tumor immune response due to prophylactic nodal irradiation. Several preclinical data from TDLN irradiation support these findings. First, some studies highlighted the key role of TDLNs in the anti-tumor immune response (33, 34). Indeed, Dammeijer et al. demonstrated that in the context of immune checkpoint inhibitors, TDLNs contribute to the anti-tumor effects by generating progenitor-exhausted T cells that seed the tumor (33). Furthermore, they showed that PD-1/PD-L1 interactions in TDLN, but not in the tumor, correlate with prognosis in melanoma patients. Marciscano et al. underlined the fact that irradiation of TDLNs restrained the adaptive immune response when stereotactic radiation and immunotherapy were associated (16). A decrease in tumor-infiltrating immune cell density such as CD8+ T cells and attenuation of chemokines associated with T-cell chemoattraction could explain this phenomenon. Similarly, Buchwald et al. found a proliferation of tumor-specific CD8+ T cells in TDLNs following tumor radiotherapy without treatment of lymph nodes (17). More recently, Darragh et al. showed that ENI to a dose of 8 Gy × 3 could disrupt the local and systemic anti-tumor response following combined primary head and neck tumor radiation (3 × 8 Gy) and immunotherapy (anti-CD25) mainly through a decrease in tumor antigen-specific T-cell priming in TDLNs and consequently decrease in circulating antigen-specific T cells (both CD4+ and CD8+) and infiltration into the tumor microenvironment (18).

All this evidence suggests that ENI is probably not the optimal strategy when combining radiotherapy and IO. While the PACIFIC trial in NSCLC is the only phase III trial to have shown a benefit of the adjunction of immune checkpoint inhibitors (ICIs) to chemoradiotherapy for locally advanced disease, the JAVELIN trial and the PEMBRORAD trial in locally advanced head and neck cancer assessing the adjunction of avelumab and pembrolizumab to chemoradiotherapy, respectively, failed to demonstrate any improvement in outcome (35, 36), nor did the KEYNOTE-412 with pembrolizumab in head and neck cancer (NCT03040999) and the CALLA trial with durvalumab in cervical cancer (NCT03830866), according to recent unpublished data (37, 38). One of the key differences between the PACIFIC trial and the other negative trials is the absence of extended ENI in the former, while it was systematically used in the latter. Therefore, the sparing of uninvolved TDLNs during the planning of radiotherapy in the context of immunotherapy could be a promising approach to optimize such a therapeutic association, along with other approaches such as margin reduction, hypofractionation, or alternative radiotherapy techniques including FLASH radiotherapy (39).

We also performed an exploratory analysis to establish a dose cutoff that should not be exceeded in NITDLN. In univariable analysis, no dose cutoff to NITDLNs was correlated with outcomes, possibly due to the lack of power in our study. Interestingly, we found that the median (incidental) dose to NITDLNs in the NILN-R− group was 23.2 Gy. This dose obtained with IMRT is lower than the incidental dose delivered to NITDLNs (approximately 40 Gy) with 3D-CRT (32). This finding underlines the fact that IMRT can achieve better TDLN radiation dose-sparing in order to obtain a stronger synergic effect when combined with IO.

Moreover, our analysis of a modern homogeneous cohort treated with CCRT and consolidation IO seems to confirm the benefit of dose reduction to circulating immune cells as suggested by Ladbury et al. in the pre-immunotherapy era (11). At the 6.3-Gy cutoff, EDRIC was a prognostic factor for PFS (≤6.3 *vs.* >6.3 Gy, p = 0.049) in univariable analysis. In multivariable analysis, there was only a trend for significance (EDRIC > 6.3 Gy: HR 3.03, p = 0.093). In addition, a recent retrospective study found similar results in a cohort of 100 patients with locally advanced NSCLC treated with IO consolidation (15). Nevertheless, McCall et al. used the equation developed by Jin et al. by considering uniform body volume between patients (10, 15), while we used the model developed by Ladbury et al. with the incorporation of MBD instead of integral total dose divided by 62 × 10^3^. The exploration of ALC at three different times was necessary to better appreciate our EDRIC data. We found that ALC ≤ 500/mm^3^ at durvalumab initiation was a poor prognostic factor for PFS in univariable analysis (HR 2.69, 95% CI 1.12–6.46, p = 0.021). Nadir ALC was also an important prognostic factor, as patients who did not experience G3/4 lymphopenia had better PFS. These results are consistent with the findings from Friedes et al. (14). Nonetheless, except for age, baseline ALC, and ALC at the end of CCRT, no clinical/dosimetric data were associated with lymphopenia ≤ 500/mm^3^ at IO initiation in our cohort, and no clinical/dosimetric correlation could be established with the LVR. Especially, neither NILN-R+ nor EDRIC was associated with lymphopenia in this cohort, perhaps because we did not consider the dose to large vessels in the model. Indeed, Cho et al. found a correlation between dose to large vessels and lymphopenia (13). Moreover, the monitoring of tumor-specific subpopulations of lymphocytes could not be assessed. For these reasons, we cannot rule out that the impact of radiation to NITDLNs on the outcome is correlated with circulating tumor-specific lymphocytes *via* a decrease in tumor antigen-specific T-cell priming.

We also explored the impact of radiation dose on other iOARs on outcomes. Dose to thoracic vertebrae was not associated with worse outcomes or lymphopenia. Doses to the spleen were very low in our cohort, so no correlation could be established.

This study has some limitations mainly due to its retrospective nature and its small cohort size. Multiple comparisons have been performed, which can inflate the alpha risk and the likelihood of type I error. However, due to the exploratory nature of our study, no adjustments were made for multiple comparisons, and all p-values and confidence intervals were shown to allow readers to interpret the results themselves according to the number of tests performed. Therefore, our results are exploratory and need to be confirmed in a larger cohort. However, notably, the single-center design guaranteed homogeneity in radiotherapy techniques and follow-up.

## Conclusion

In conclusion, we found that prophylactic irradiation of at least one NITDLN was a strong independent factor for worse PFS in patients treated with consolidation immunotherapy following CCRT for locally advanced NSCLC. Moreover, we confirmed the impact of lymphopenia and irradiation of immune cells (EDRIC) on outcomes in this population. These findings lend weight to the idea that modern radiotherapy techniques should spare host immune structures and especially NITDLNs when combining radiotherapy and immunotherapy for locally advanced disease.

## Data availability statement

The raw data supporting the conclusions of this article will be made available by the authors, without undue reservation.

## Ethics statement

The studies involving human participants were reviewed and approved by Institutional review boards of Toulouse Cancer Institute. Written informed consent for participation was not required for this study in accordance with the national legislation and the institutional requirements.

## Author contributions

Conceptualization, JK and CP; methodology, JK and CP; formal analysis, CP, LC, AL, and JK; investigation, all; data curation, CP; writing—original draft, CP and JK; writing—review and editing, all; supervision, JK. All authors contributed to the article and approved the submitted version.
