# Supplementary material for: Impact of radiation on host immune system in patients treated with chemoradiotherapy and durvalumab consolidation for unresectable locally advanced non-small cell lung cancer
Source: Front Oncol. 2023 Jun 16;13:1186479. doi: 10.3389/fonc.2023.1186479 (PMC10313116; doi:10.3389/fonc.2023.1186479)
Supplement: Supplementary file 1 [file Table_1.docx]

**Suppl. Table 1** – Assocation between lymphopenia at durvalumab initiation and clinical/dosimetric/biological data

|  | ALC at durvalumab initiation ≤ 500/mm^3^  (N=11) | ALC at durvalumab initiation > 500/mm^3^  (N=39) | p-value |
| --- | --- | --- | --- |
| Age at initial diagnosis  (median, range) | 68 (59 – 73) | 59 (36 – 75) | 0.017 |
| Sex  Male  Female | 91%  9% | 72%  28% | 0.257 |
| Chemotherapy regimen  Carboplatin + Vinorelbine  Cisplatin + Vinorelbine | 46%  54% | 39%  61% | 0.736 |
| Radiation total dose  (median Gy), (range) | 66 Gy  (55 – 66) | 66 Gy  (55 – 66) | 0.814 |
| Volume of PTV  (median cm3) , range) | 428.9 (212.2 – 551.2) | 305.8 (114.1 – 1284) | 0.319 |
| Volume of tumor GTV  (median, cm3) (range) | 70.2 (2.4 – 127.9) | 45.8 (0.4 – 837.5) | 0.923 |
| Mean heart dose  (median, Gy) (range) | 11.2 (2.1 – 16.6) | 8.9 (0.8 – 18) | 0.699 |
| Mean lung dose  (lung minus PTV)  (median, Gy) (range) | 12.9 (10.2 – 16.5) | 12 (5.6 – 19.2) | 0.223 |
| Mean dose to T1-T12  (median, Gy) (range) | 13.6 (8.2 – 16.4) | 10.9 (2.9 – 22.6) | 0.814 |
| Mean body dose  (median, Gy) (range) | 6.9 (5.2 – 9.1) | 7.3 (2.7 – 12) | 0.752 |
| EDRIC  (median, Gy) (range) | 7.6 (5.8 – 9.4) | 7.8 (2.8 – 11.6) | 0.981 |
| EDRIC  ≤ 6.3 Gy (n, %)  > 6.3 Gy (n, %) | 2 (18%)  9 (82%) | 12 (31%)  27 (69%) | 0.705 |
| Volume NITDLN  (median, cm^3^) (range) | 26.3 (0 – 79.3) | 26.6 (0 – 132.1) | 0.778 |
| Mean dose to NITDLN  (median, Gy) (range) | 27.4 (10.5 – 64.3) | 28.9 (3.1 – 58.9) | 0.929 |
| NILN-R+  Yes  No | 3 (27%)  8 (73%) | 8 (21%)  31 (79%) | 0.688 |
| ALC at baseline  (median, mm^3^) (range) | 1400 (628 – 2300) | 1800 (770 – 3060) | 0.01 |
| ALC at end of CCRT  (median, mm^3^) (range) | 388 (130 – 700) | 530 (290 – 1500) | 0.002 |
| Lymphocyte variation rate  (median, %) (range) | -74.4 (-84.3 ; -61.1) | -71.4 (-84.6 ; -26.7) | 0.085 |

Abbreviations: NILN-R+ : inclusion of at least one non-involved tumor draining lymph node ; NITDLN : non-involved tumor draining lymph node ; EDRIC : estimated dose to immune cells ; ALC : absolute lymphocyte count.
