# Supplementary material for: Impact of radiation on host immune system in patients treated with chemoradiotherapy and durvalumab consolidation for unresectable locally advanced non-small cell lung cancer
Source: Front Oncol. 2023 Jun 16;13:1186479. doi: 10.3389/fonc.2023.1186479 (PMC10313116; doi:10.3389/fonc.2023.1186479)
Supplement: Supplementary file 2 [file Table_2.docx]

**Suppl. Table 2** – Spearman correlation between LVR (Lymphocyte Variation Rate) and continuous variables.

|  | Spearman coefficient for LVR | p-value |
| --- | --- | --- |
| Age at diagnosis | -0.073 | 0.612 |
| Volume of PTV (cm^3^) | -0.173 | 0.231 |
| Volume of NITDLN (cm^3^) | -0.221 | 0.124 |
| Mean dose to NITDLN (Gy) | 0.244 | 0.119 |
| V10Gy to NITDLN (%) | 0.264 | 0.091 |
| V20Gy to NITDLN (%) | 0.235 | 0.134 |
| V30Gy to NITDLN (%) | 0.225 | 0.152 |
| V40Gy to NITDLN (%) | 0.216 | 0.169 |
| V50Gy to NITDLN (%) | 0.232 | 0.140 |
| Mean dose to T1-T12 (Gy) | -0.163 | 0.257 |
| EDRIC (Gy) | -0.199 | 0.166 |
| Mean total body dose (Gy) | -0.194 | 0.177 |
